# Cardiac Rehabilitation in the WHO Eastern Mediterranean Region: A Scoping Review with a Saudi Arabia–Focused Synthesis

**DOI:** 10.3390/jcm15124413

**Published:** 2026-06-07

**Authors:** Wael Alghamdi

**Affiliations:** Nursing Faculty, Al-Baha University, Al-Baha 65779, Saudi Arabia; waelalghamdi@bu.edu.sa

**Keywords:** secondary prevention, coronary artery disease, myocardial infarction, coronary artery bypass grafting, health services accessibility, referral pathways, patient participation, implementation barriers

## Abstract

Cardiac rehabilitation (CR) is underused globally, and evidence on its availability and delivery in the World Health Organization Eastern Mediterranean Region (WHO EMR) remains fragmented. This scoping review mapped evidence on cardiac rehabilitation (CR) across countries of the World Health Organization Eastern Mediterranean Region (WHO EMR), including service availability, delivery models, participation pathways, and barriers and enablers, with focused consideration of Saudi Arabia. Studies published from 1 January 2000 to 14 January 2026 were searched in MEDLINE, Scopus, Web of Science Core Collection, CINAHL, Embase, and the WHO Index Medicus for the Eastern Mediterranean Region. Data were synthesised descriptively and narratively in accordance with scoping review methodology. Twenty-five studies met the inclusion criteria, covering eight WHO EMR countries and one regional audit. Evidence was unevenly distributed, centre-based outpatient CR predominated, and losses were most evident at referral and enrolment stages. CR evidence across the WHO EMR remains fragmented and uneven. Stronger referral pathways, greater service capacity, and flexible delivery models are needed, including in Saudi Arabia.

## 1. Introduction

Cardiovascular disease remains a major contributor to morbidity and mortality worldwide [1]. Consequently, effective secondary prevention is a prime goal in reducing long-term disability, overall disease burden, and the recurrence of cardiovascular events [1,2]. A major component of current programmes for the management of coronary artery disease and recovery after major cardiac events or revascularisation is exercise-based cardiac rehabilitation (CR). Cardiac rehabilitation is not simply an exercise programme; it is a multifaceted approach that combines physical exercise with comprehensive patient education, identification and modification of risk factors, and behavioural support [2,3]. Evidence from a variety of sources indicates that CR has been associated with improved survival, reduced hospitalisations and recurrent cardiovascular events, and better health-related quality of life [3,4].

Despite such encouraging evidence, levels of CR participation remain low across the world [2,5]. From referral through enrolment on programmes and continued participation, gaps persist across the care pathway [2,6]. International mapping studies show that CR services are not widely available and, where they are available, are often insufficient for the populations they are intended to serve [5]. The crucial issue around CR, therefore, is less its effectiveness than the practical challenges involved in implementing it, particularly limitations in access, service organisation, and system-level delivery [2,6]. Strategies such as systematic referral processes and alternative modes of delivery, including remote, home-based, and hybrid models, have been proposed to address barriers such as long travel distance and limited service reach; however, equitable access remains difficult to achieve [7,8].

These challenges are particularly relevant to the 22 countries of the World Health Organization Eastern Mediterranean Region (WHO EMR) [9]. Across the region, there is a substantial burden of cardiovascular disease, accompanied by wide differences in healthcare systems, workforce capacity, and access to specialised rehabilitation services [1,9]. Such heterogeneity results in wide variation in the availability of CR and in patients’ opportunities to engage with it [10,11]. At the same time, the existing evidence on CR in the EMR remains limited and fragmented, and current reports suggest that provision of services is insufficient relative to need [11,12].

There is wide methodological diversity in the literature on this subject, encompassing programme evaluations, observational studies, registry analyses, surveys, qualitative investigations, and intervention studies [13,14]. Given the heterogeneity of evidence on CR availability, delivery models, participation metrics, and implementation barriers across WHO EMR countries, a scoping review was appropriate to map how the topic has been studied and to identify service and research gaps [13,15].

Accordingly, this review aimed to map the availability and geographic distribution of CR services across countries of the World Health Organization Eastern Mediterranean Region (WHO EMR), characterise delivery models, programme characteristics, and multidisciplinary involvement, and summarise reported referral and participation metrics, including enrolment, adherence, completion, and attrition where available. It also aimed to identify multilevel barriers and enablers to CR implementation and participation, and to provide a Saudi Arabia-focused synthesis highlighting context-specific evidence gaps and priorities for service development and future research. The Saudi Arabia-focused synthesis was planned in the registered protocol. It was included because six of the included studies were from Saudi Arabia, and the findings may be useful for national CR service planning.

## 2. Methods

### 2.1. Study Design

The purpose of this study was to characterise the contemporary evidence on CR across WHO EMR countries, with particular attention to Saudi Arabia as a planned component of the registered protocol [13,14]. The scoping methodology was chosen in light of the heterogeneity of the existing literature, which encompasses observational studies, service evaluations, surveys, qualitative research, and intervention-based designs [14]. Rather than seeking to generate pooled effect estimates, the review aimed to describe the breadth, nature, and distribution of evidence, as well as how CR has been reported in relation to service availability, delivery models, referral and participation pathways, and barriers and enablers to participation and completion [13,14]. This national synthesis was intended to place the Saudi evidence within the regional map and to identify context-specific service gaps and planning priorities. The protocol was registered with the International Platform of Registered Systematic Review and Meta-analysis Protocols on 15 January 2026 (INPLASY202610048), before the formal database searches began on 20 January 2026; reporting followed the Preferred Reporting Items for Systematic Reviews and Meta-Analyses extension for Scoping Reviews (PRISMA-ScR) and the completed PRISMA-ScR checklist is provided as Supplementary Material S1 [16].

### 2.2. Eligibility Criteria

The definition of the relevant criteria was in line with the Participants–Concept–Context framework [14,17]. The target sample was adults with a history of coronary artery disease, myocardial infarction, and/or coronary artery bypass grafting. Eligibility was confined to studies reporting CR among participants with these conditions; studies focused exclusively on those aged under 18 years were excluded.

The concept related to how exercise-based CR has been implemented. Qualifying research involved reporting on the availability and geographic distribution of CR services, referral pathways, multidisciplinary involvement, and participation-related outcomes, including uptake, enrolment, adherence, completion, and attrition [17]. Research relating to centre-based, home-based, hybrid, or telehealth/digital CR was deemed relevant if a clearly defined programme or service was involved. Lifestyle advice and non-programmatic prevention were excluded [17].

The context was confined to WHO EMR countries. Research from a single EMR country or broader studies was included where EMR-specific data were available. In line with the review objectives, Saudi Arabia–specific findings were examined to create a focused regional synthesis [17]. Eligible sources consisted of peer-reviewed primary research using quantitative, qualitative, or mixed-methods approaches, together with programme evaluations and registry-based or retrospective studies. Review articles, editorials, commentaries, conference abstracts without full text, and non-peer-reviewed material were deemed ineligible [17].

### 2.3. Information Sources and Search Strategy

To access regional research sources, the literature search was conducted in MEDLINE (via PubMed), Scopus, Web of Science Core Collection, CINAHL, Embase, and the WHO Index Medicus for the Eastern Mediterranean Region (IMEMR) [14]. The publication date range was from 1 January 2000 to 14 January 2026. No language restrictions were applied at the search stage. No potentially eligible non-English full-text articles requiring translation were identified.

Database searches were complemented by screening the reference lists of included studies, and forward citation tracking was conducted for key articles. The search used a combination of controlled vocabulary and free-text terms relating to CR in WHO EMR countries, secondary prevention, service implementation, and participation measures and determinants [14]. The search was deliberately focused on CR service delivery, participation, and implementation in WHO EMR countries, in line with the review objectives. The search strategies were tailored to each database’s indexing framework and are listed in Supplementary Material S2 [16].

### 2.4. Study Selection

Records were deduplicated in Zotero using its duplicate-detection function and checked before screening in Covidence [16]. Study selection was led by the author using the predefined eligibility criteria. A second reviewer with health research experience independently verified all title/abstract screening decisions (108/108, 100%) and all full-text eligibility decisions (88/88, 100%). Any uncertainties were resolved through discussion and rechecking against the predefined eligibility criteria. Formal inter-reviewer agreement statistics were not calculated for this verification step. Reasons for excluding full-text articles were recorded, and the overall selection process was summarised using a PRISMA-ScR flow diagram [16].

### 2.5. Critical Appraisal

In line with scoping review objectives, which focus on charting the breadth and characteristics of the evidence rather than creating pooled estimates, no formal methodological quality assessment was conducted. However, key methodological characteristics of the included studies were recorded and considered in the interpretation of the findings [14].

### 2.6. Data Charting and Extraction

A structured charting form, specifically created for the study, was used to extract data and was piloted on a subset of studies before full implementation. Data charting was conducted by the author. A second reviewer independently verified the charted data for all included studies (25/25, 100%). Any uncertainties were resolved through discussion and rechecking against the source articles and the predefined charting framework. Formal inter-reviewer agreement statistics were not calculated for this verification step. Extracted variables consisted of bibliographic information, including author, year, country, and design, as well as population and setting features and details on the study objectives [14].

### 2.7. Data Synthesis

In order to synthesise the data relating to evidence on CR within the WHO EMR, a descriptive and narrative approach was employed. The quantitative findings were recorded as frequencies, proportions, and ranges; meta-analysis was not conducted. The domains around which the synthesis was organised were:

- availability and geographic distribution of CR services
- delivery models and programme characteristics
- referral pathways and participation-related outcomes, including enrolment, adherence, completion, and attrition where reported
- barriers and enablers influencing implementation and participation

Tabular summaries and narrative synthesis were used to present the findings for the overall region, while additional attention was given to data on Saudi Arabia in order to detail context-specific gaps and service implications [14].

## 3. Results

### 3.1. Study Selection

**Figure 1:**
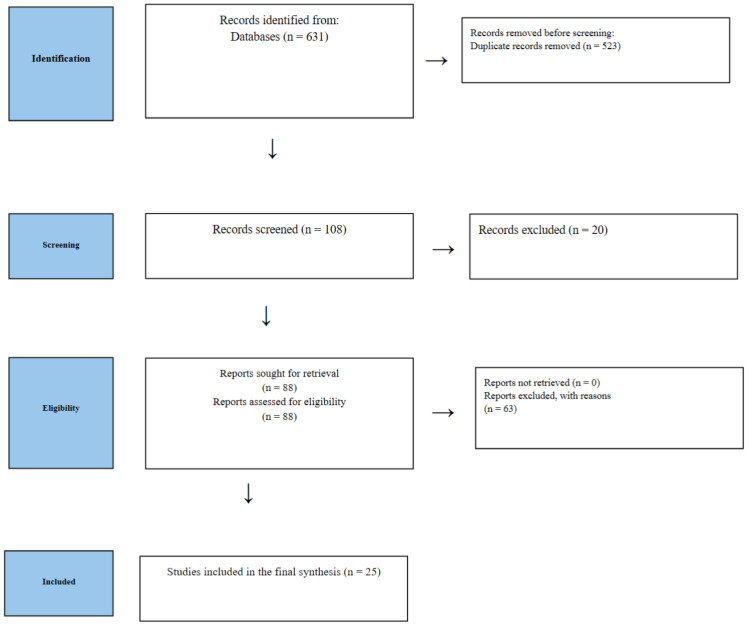
PRISMA-ScR flow diagram of the study selection process.

A total of 631 records were retrieved from the database searches. After removal of 523 duplicate records, 108 titles and abstracts were screened, of which 20 were excluded. Full-text eligibility was assessed for 88 reports, and 63 were excluded for prespecified reasons. The final synthesis included 25 studies, as shown in the PRISMA-ScR flow diagram (Figure 1).

### 3.2. Overview of the Included Evidence

The final dataset comprised 25 studies from various countries within the WHO EMR, with an uneven distribution of contributions, the largest being from Iran (*n* = 8) and Saudi Arabia (*n* = 6) (Table 1). The majority of the studies were observational, cross-sectional, or survey-based, whereas randomised trials, qualitative research, registry-based analyses, audits, and programme reports were less frequent. In general, the findings indicate that the evidence base was heterogeneous, with individual countries displaying wide differences in the quantity and type of available data [11,12,18,19,20,21,22,23,24,25,26,27,28,29,30,31,32,33]. Detailed characteristics of the 25 included studies are provided in Supplementary Table S1; Table 2, Table 3 and Table 4 summarise selected synthesis domains and are not intended to replace the comprehensive study-characteristics table.

### 3.3. Availability and Geographic Distribution of CR Evidence

Saudi Arabia, Iran, Qatar, the United Arab Emirates, Pakistan, and Algeria provided more comprehensive programme- and service-level descriptions, whereas data from Lebanon and Morocco were more limited and consisted mainly of survey-based studies focusing on awareness, preferences, attitudes, referral barriers, and implementation challenges, rather than detailed descriptions of established CR services. An EMR audit showed that CR is available only in a subset of countries, with programme density and capacity remaining low relative to population needs in those settings (Table 2). Overall, most detailed programme-level data were generated by only a few countries, as illustrated in Table 1.

**Table 2:** Programme characteristics and delivery models.

Study/Country	Delivery Model	Key Programme Characteristics	Team/Service Features	Main Relevance to Review
EMRO regional audit (2019)	Mixed regional picture	Assessed availability, density, capacity, and delivery of CR across EMRO	System-level mapping	Demonstrated limited regional availability and low capacity
Saudi Arabia: post-CABG RCT (2022)	Home-based vs. outpatient-based vs. usual care	Compared structured home-based and outpatient CR after CABG	Rehabilitation intervention	Showed home-based CR was effective and may sustain gains
Saudi Arabia: home-based CR trial (2012)	Home-based	Education, follow-up phone calls, workshops, family involvement	Multicomponent home programme	Demonstrated feasibility and benefit of home-based CR
UAE: Abu Dhabi registry (2023)	Centre-based outpatient	Exercise-based outpatient CR registry from 2015–2022	Physical therapist-led programme	Provided direct Gulf programme description and completion factors
Qatar first CR programme (2021)	Centre-based outpatient	Sole national CR programme with engagement and outcome data	Established programme service	Demonstrated feasibility and high completion in a national service
Qatar hybrid phase II programme (2023)	Hybrid	Hybrid CR delivery during COVID-19 with safety and cost data	Programme adaptation model	Showed hybrid CR was feasible, safe, and lower cost
Iran provincial audit (2023)	Mainly centre-based, some home-based	National/provincial mapping; median supervised dose 14 sessions; about one-third offered home-based services	Mostly multidisciplinary	Documented programme distribution and service characteristics
Iran registry experience (2023)	Centre-based, home-based, hybrid	Phased CR workflow with long-term registry follow-up	Registry-supported multidisciplinary model	Highlighted registry-based quality improvement and model flexibility
Iran Yazd programme report (2019)	Multi-phase centre-based with alternatives	Four phases; outpatient phase 36 sessions over 3 months	Multidisciplinary team	Provided detailed service-level description in a developing-country context
Pakistan local experience (2012)	Centre-based outpatient	6-week outpatient CR after AMI/CABG/PCI	Service access-oriented	Identified attendance/completion patterns in routine practice
Pakistan qualitative home-based design study (2025 VOR)	Contextual home-based model	Explored patient needs to inform locally tailored home-based CR	Patient-informed model design	Supported need for contextualized home-based CR
Pakistan MCard trial (2022)	Digitally supported/mHealth-augmented	mHealth added to standard post-ACS care	Technology-supported care	Suggested scalable low-cost extension of CR services
Algeria first experience (2008)	Early centre-based programme	First Algerian CR centre and early outcomes in coronary patients	Early implementation report	Demonstrated initial feasibility of CR establishment

This table focuses on studies that described CR delivery pathways, programme/service architecture, or intervention characteristics relevant to the review objectives. Delivery models were categorized as centre-based, home-based, hybrid, or digitally supported in accordance with the protocol.

### 3.4. Delivery Models and Programme Characteristics

Table 2 indicates the range of CR delivery models across the included studies; these encompassed centre-based, home-based, hybrid, and digitally supported approaches. The programmes most frequently described were centre-based outpatient programmes, with most of the reported studies coming from Iran, Qatar, the United Arab Emirates, Pakistan, and Algeria [11,12,21,22,24,25,26,29]. These reports and audits provided useful insights into how services were organised. Saudi Arabia provided most of the information on home-based CR, especially in relation to patients after coronary artery bypass grafting; data from Iran and Pakistan were also included [25,26,31,33,34]. Qatar provided information on hybrid models, while Iran contributed information on registry- or service-based pathways. A Pakistani mHealth-enhanced model described digitally supported delivery [27]. Although there was variation across settings, the core components tended to include supervised or prescribed exercise, patient education, risk factor management, follow-up support, and multidisciplinary involvement [12,20,21,22,24,25,26,27,29,31,34]. Teams also tended to be multidisciplinary, with an Abu Dhabi centre being described as having a physical therapist-led outpatient CR programme [21]. In general, the findings reveal growing interest in flexible delivery models in order to help address capacity constraints within the region [11,20,22,24,25,26,27,31].

### 3.5. Referral, Uptake, Adherence, Completion, and Attrition

As illustrated in Table 3, the studies reveal inconsistency in participation-related metrics; there were variations in the definitions of referral, uptake, enrolment, adherence, completion, and attrition, which restrict straightforward comparison. Despite this, the available studies suggest that losses may be concentrated early in the CR pathway, particularly at referral and enrolment stages [12,21,25,26,35].

**Table 3:** Participation metrics across key studies.

Country/Study	Referral	Uptake/Enrolment/Attendance	Completion/Attrition	Notes
SAUDI ARABIA: POST-PCI PATIENT SURVEY (2024)	10.6% referred	36.4% of those referred attended	Not fully reported	Home-based CR preferred by 58.7%
IRAN: WEST OF IRAN POST-CABG (2014)	44.6% referred	18.7% enrolled	16.5% completed	Systematic referral improved participation
IRAN: YAZD PROGRAMME REPORT (2019)	60% referral by inpatient CR team	Participation 6.9%; enrolment 55%	57% completed	Only CR programme in Yazd province
IRAN: KERMANSHAH COMPLETION STUDY (2015)	Not reported	CR attendees analyzed	49% completed; 51% dropped out	Failure to complete linked to social/psychological factors
QATAR FIRST CR PROGRAMME (2021)	Not reported	77.6% of prescribed sessions attended	81.2% completed	High engagement within established service
QATAR HYBRID PHASE II (2023)	Not reported	51 enrolled in hybrid model	84.3% completed	No major adverse events reported
PAKISTAN LOCAL EXPERIENCE (2012)	Not reported	36.2% enrolled and attended	73.4% completed >6 weeks	Attendance associated with easier access
UAE ABU DHABI REGISTRY (2023)	Not uniformly reported	Registry attendees described	Completion pragmatically defined as ≥10 sessions	Completion associated with geography, BMI, depression

Definitions varied across studies; metrics are presented descriptively and should not be interpreted as directly comparable.

Among Saudi Arabian patients who had undergone percutaneous coronary intervention, CR referral was only 10.6%, with just 36.4% of those referred attending (Table 3) [30]. In contrast, referral in an Iranian programme was reported at 60%, whereas participation was 6.9% [25]. These figures, however, were based on study-specific definitions, and the denominators and definitions differed across studies, limiting direct comparison. When patients had already entered established programmes, enrolment and completion estimates tended to be higher; however, the consistency of these estimates varied according to the denominators and reporting approaches used across studies.

In general, the data suggest that the more important influences on participation may lie in service access, referral systems, and early enrolment pathways rather than in within-programme retention once patients have entered CR. Given the variability in reporting across studies, however, these findings should be interpreted with caution.

### 3.6. Barriers and Enablers

Barriers to CR were identified across patient, provider, organizational/health-system, and policy and financing domains (Table 4). Patients often reported long travel distance, transport costs, work or family pressures, low awareness, limited motivation, comorbidities, and psychological factors such as anxiety or depression as barriers to participation [11,23,30,33,36,37]. Provider-level barriers included limited knowledge of CR and its potential benefits, inconsistent referral practices, and shortages of trained staff [11,18,19,23,28,36]. At the organisational and health-system level, barriers included limited programme availability, geographic maldistribution of services, concentration in urban centres, fragmented referral pathways, and workforce or resource constraints [11,18,19,23,28,30,36]. Policy and financing barriers included weak insurance coverage, insufficient funding, lack of structured national implementation frameworks, and limited strategic support [11,23,30,36].

**Table 4:** Multi-level matrix of barriers and enablers.

Level	Recurring Barriers	Recurring Enablers
**Patient level**	Transport burden, long travel distance, out-of-pocket costs, low awareness, low motivation, anxiety/depression, comorbidities, work or family constraints	Home-based CR, hybrid models, tailored education, telephonic/remote support, family involvement
**Provider level**	Limited CR knowledge, inconsistent referral practices, low awareness of CR phases/benefits, insufficient trained personnel	Provider education, stronger endorsement by specialists, simplified/automated referral systems, increased professional exposure to CR
**Organisational/health-system level**	Limited number of CR centres, geographic maldistribution, concentration in major cities/capitals, fragmented referral pathways, workforce/resource shortages	Multidisciplinary service development, wider regional coverage, programme standardisation, registry-based monitoring, flexible delivery pathways
**Policy/financing level**	Weak insurance coverage, funding limitations, lack of structured national implementation pathways, insufficient strategic support	Leadership support, reimbursement reform, Arabic/local guidance, institutional coordination, service scale-up planning

Themes were mapped to the four implementation domains prespecified in the protocol: patient, provider, organisational/health-system, and policy/financing.

On the positive side, the included studies pointed to several enablers, including home-based and hybrid CR models, tailored patient education, telephonic or remote follow-up, improved provider awareness, stronger specialist endorsement, simplified referral pathways, multidisciplinary programme development, registry-based monitoring, service standardisation, leadership engagement, and reimbursement reform [11,19,23,30,33,36,37]. Collectively, these findings indicate that improving CR uptake and implementation in the EMR will require coordinated action across multiple levels rather than interventions focused solely on patients (Table 4) [11,36].

### 3.7. Saudi Arabia–Focused Synthesis

Saudi Arabia was prominently represented in the included literature, with evidence spanning policy, provider, patient, and trial perspectives. The Saudi Arabia-specific evidence is summarised separately in Table 5 to distinguish national findings from the broader EMR synthesis. Taken together, the Saudi evidence suggests that the core challenge is not uncertainty about the value of cardiac rehabilitation, but the limited translation of that value into routine and accessible care pathways (Table 5). Across the Saudi evidence base, CR was consistently viewed as beneficial; however, access remained constrained by limited programme availability, uneven geographic coverage, underdeveloped referral systems, and workforce- and guidance-related limitations [18,19,20,30,31,36].

Provider- and policy-focused studies highlighted structural barriers, including the absence of local CR programmes in many settings, fragmented referral pathways, limited awareness of CR processes and benefits, shortages of trained personnel, and broader implementation and funding challenges [18,19,36]. Patient-level evidence following percutaneous coronary intervention similarly suggested that low referral was accompanied by limited attendance among those referred, while patients from more remote areas appeared particularly receptive to home-based CR [30]. Evidence from Saudi post–coronary artery bypass grafting trials further supports the feasibility and clinical value of home-based CR, with outcomes broadly comparable to outpatient models in the reported settings [20,31]. Viewed collectively, these findings suggest that, in Saudi Arabia, recognised need and generally positive stakeholder attitudes have not yet translated into equitable routine access, and that flexible delivery pathways may represent a particularly relevant strategy for future service development.

**Table 5:** Saudi Arabia-specific cardiac rehabilitation evidence included in the review.

Study/Saudi Evidence Area	Design/Population	CR Focus or Programme Type	Referral/Uptake/Completion Metrics	Main Findings/Barriers	Key Limitations
**Saudi policymakers qualitative study [36]**	Qualitative interpretive descriptive study; 9 policymakers/leaders	Policy, implementation, and uptake of CR	Not applicable	Identified system-level barriers and enablers to CR uptake, including implementation, service organisation, and broader planning issues	Small stakeholder sample; reflects policy and leadership perspectives rather than patient- or programme-level outcomes
**Western Saudi outpatient CR barriers study [18]**	Cross-sectional survey; 141 respondents	Establishing outpatient/phase III CR	Not fully reported	Highlighted limited CR availability, low awareness, workforce constraints, and lack of local guidance	Cross-sectional survey; regional focus; findings may not represent all Saudi settings
**Saudi cardiologists KAP/referral survey [19]**	Cross-sectional online survey; 140 cardiologists	Knowledge, attitudes, practices, and referral barriers after PCI	Low/variable referral reported	Showed moderate-to-good CR knowledge but inconsistent referral practices and ongoing referral barriers	Self-reported data; limited to cardiologists’ perspectives; referral behaviour may be affected by response bias
**Saudi post-PCI patient barriers survey [30]**	Cross-sectional telephone survey; 104 post-PCI patients	Enrolment barriers and secondary prevention adherence	10.6% referred; 36.4% of referred patients attended; completion not fully reported	Demonstrated very low referral and attendance; 58.7% preferred home-based CR	Self-reported referral and attendance; cross-sectional design; limited completion data
**Saudi post-CABG RCT [31]**	Three-arm single-blind RCT; 82 randomised post-CABG patients	Home-based vs. outpatient-based CR vs. usual care	Not directly comparable with service-level referral or uptake metrics	Home-based CR was effective and may sustain benefits compared with usual care/outpatient pathways in the trial setting	Trial setting; selected post-CABG population; generalisability to routine service implementation may be limited
**Saudi home-based CR RCT [20]**	RCT; 49 post-CABG men	Home-based CR vs. usual care; education, follow-up calls, workshops, and family involvement	Not fully reported	Demonstrated feasibility and benefit of a multicomponent home-based CR programme	Small sample; male-only post-CABG population; limited service-level implementation data

This table summarises the Saudi Arabia-specific studies included in the review. Themes were mapped to the four implementation domains prespecified in the protocol: patient, provider, organisational/health-system, and policy/financing.

## 4. Discussion

Across EMR countries, the central pattern was that inconsistent uptake of CR appeared to reflect access and implementation barriers rather than uncertainty about its clinical value. This pattern is consistent with global CR availability studies and the EMR-specific audit, which show that CR remains unevenly available and that service capacity is often insufficient for population needs. Because participation metrics were defined and reported heterogeneously, these findings should be interpreted cautiously; nevertheless, the available data indicate that fragmented referral and enrolment processes may contribute to low initial uptake.

Evidence on early participation pathways supports this pattern, with losses appearing more pronounced before patients entered CR programmes than after enrolment [25,30]. This is consistent with broader international evidence showing that CR remains underutilised even where its benefits are well established [38]. When structured or automatic referral pathways are embedded in routine practice, uptake tends to improve compared with physician-dependent referral [39]. Low referral and enrolment rates therefore likely reflect not only patient-related factors, but also limitations in patient identification, referral pathways, and early integration into CR services.

Across EMR studies, there was increasing interest in hybrid, home-based, and digitally supported CR models [22,27,34]. In many settings, these models seem to address limited access to services concentrated in urban centres. These models may therefore extend CR delivery rather than replace established centre-based services [22,34]. The 2023 Cochrane review supports home-based CR for selected patients, while suggesting that flexible models should complement, not replace, centre-based care [8,40].

Findings from the Saudi literature provide a useful national example of how broader EMR implementation challenges may operate in practice [20,36]. Across the included Saudi studies, CR was consistently recognised as beneficial; however, this recognition had not yet translated into equitable routine access. Instead, the available evidence points to an implementation gap characterised by limited programme availability, underdeveloped referral pathways, and workforce-related constraints [30,36]. This strengthens the case for greater use of home-based and other flexible CR models, not as substitutes for established centre-based services, but as pragmatic approaches to geographic and organisational barriers that continue to limit access to CR [20,30].

The findings have several implications for policy and practice. If CR participation is to improve, focus should not be restricted to patient-level factors; instead, policy and service planning should address referral pathways, provider awareness, workforce development, service availability, and organisational support [30,36]. Using the Consolidated Framework for Implementation Research (CFIR) as a practical lens, the findings indicate that CR implementation in the WHO EMR is shaped by the interaction between patients’ circumstances, service capacity, and the wider health-system environment [41]. Limited reimbursement, uneven policy support, and concentration of services in major cities may restrict access before many patients can consider participation. Within health services, workforce shortages, limited programme capacity, and inconsistent referral routines may interrupt the pathway from hospital discharge to CR enrolment. These service-level barriers may then interact with patient-level concerns, including health beliefs, psychological burden, travel difficulty, and work or family responsibilities. From a health-equity perspective, patients living outside major urban centres, those with fewer financial resources, those with lower health literacy, and those with gender-sensitive access needs may face greater difficulty reaching centre-based programmes. Flexible home-based, hybrid, and digital models may broaden access, but they require clear referral processes, follow-up, and professional supervision to avoid becoming less-supported options for patients already facing barriers.

Regardless of delivery model, CR services should retain core components such as exercise, education, risk-factor management, and behavioural support. Recent literature on CR in other cardiac populations, including atrial fibrillation, also supports the value of multidisciplinary rehabilitation beyond exercise alone [42]. Nevertheless, the current evidence base remains weighted toward observational and survey-based research, with relatively limited representation of implementation-focused evaluations, registry-based monitoring, qualitative research, and interventional designs [31,36]. This pattern suggests that, particularly in low- and middle-income settings, the field is shifting from demonstrating the value of CR to understanding how it can be implemented effectively in real-world health systems [5,43]. Future research in the EMR should therefore prioritise strategies to improve referral, enrolment, and sustained participation, especially where access remains limited.

This review has several strengths relevant to interpretation. It was conducted using a prospectively registered protocol and a clearly defined scoping methodology, which allowed a heterogeneous body of evidence to be mapped systematically. Through the inclusion of multiple study designs, including audits, programme reports, registry-based studies, surveys, qualitative research, and interventional studies, CR could be examined as both a clinical and a service-delivery intervention. Additionally, the use of structured evidence-mapping tables enabled synthesis across country, programme, participation, and implementation domains, thereby strengthening the practical and policy relevance of the findings.

The results should, however, be interpreted in light of certain limitations. First, the evidence was unevenly distributed, with Iran and Saudi Arabia contributing substantially more than other EMR countries. This uneven distribution of published evidence should not necessarily be interpreted as the absence of CR services in other EMR countries, but may also reflect limited publication, indexing, or accessible programme-level reporting. Because the search included service and implementation terms, studies reporting clinical outcomes without describing CR delivery, referral, participation, or barriers may have been missed. Second, observational and survey-based studies were more common, whereas detailed service evaluations, qualitative implementation studies, and controlled trials were less frequently represented. Third, participation-related metrics varied in both definition and reporting, including referral, uptake, enrolment, adherence, completion, and attrition, which limited straightforward comparison across studies. Fourth, although a second reviewer verified all screening decisions and charted data, the review process was primarily author-led and formal agreement statistics were not calculated. This differed from the duplicate independent screening process anticipated in the registered protocol and is therefore acknowledged as a methodological limitation. Accordingly, the possibility of selection or charting error cannot be fully excluded. Finally, as a scoping review, this study was designed to capture the breadth and nature of the available evidence rather than to generate pooled estimates or support comparative effectiveness conclusions. The findings should therefore be viewed as an overview of the current evidence landscape rather than as a basis for direct cross-context comparison.

## 5. Conclusions

This scoping review suggests that evidence on CR in the WHO EMR is growing, but geographic coverage, service access, and depth of evidence remain fragmented. The central theme in the literature is not uncertainty about the value of CR, but the limited translation of this value into effective referral pathways, accessible services, and sustained participation. Saudi Arabia reflects a similar pattern, with stakeholders recognising the need for CR while continuing to face challenges in achieving equitable access. Future priorities should include strengthening referral practices, expanding service capacity, and supporting flexible delivery models across the EMR.

## Figures and Tables

**Table 1:** Country-level evidence map.

Country/Region	No. of Included Studies	Main Evidence Types
EMRO region	1	Regional audit
Saudi Arabia	6	Qualitative, cross-sectional surveys, randomized trials
United Arab Emirates	1	Registry-based retrospective study
Qatar	2	Retrospective cohort, quality improvement
Lebanon	2	Survey/preference studies
Iran	8	Audit, registry report, programme report, cross-sectional, retrospective observational
Pakistan	3	Cross-sectional, qualitative, randomized trial
Morocco	1	Cross-sectional survey
Algeria	1	Early programme report

Counts refer to the primary country or region reported for each included study; the EMRO regional audit was counted separately.

## Data Availability

All data generated or analysed during this scoping review are included in this article and its Supplementary Materials.

## Supplementary Materials

The following supporting information can be downloaded at: https://www.mdpi.com/article/10.3390/jcm15124413/s1, Supplementary Material S1: Completed PRISMA-ScR checklist; Supplementary Material S2: Full database-specific search strategies; Supplementary Table S1: Characteristics of the included studies.
