# Supplementary material for: Cardiac Rehabilitation in the WHO Eastern Mediterranean Region: A Scoping Review with a Saudi Arabia–Focused Synthesis
Source: J Clin Med. 2026 Jun 7;15(12):4413. doi: 10.3390/jcm15124413 (PMC13300928; doi:10.3390/jcm15124413)
Supplement: Supplementary file 1 [file jcm-15-04413-s001.zip › Supplementary Material S1.pdf]

# Supplementary Material 1. Completed PRISMA-ScR Checklist

*Cardiac Rehabilitation in the WHO Eastern Mediterranean Region: A Scoping Review with a Saudi Arabia-focused Synthesis*

Note. This checklist is based on the PRISMA extension for Scoping Reviews (PRISMA-ScR). Locations are reported by manuscript section, table, figure, or supplementary file because final page numbers may change during journal production.

| Item | PRISMA-ScR section | Checklist item | Reported location | Comments / notes |
|---|---|---|---|---|
| 1 | Title | Identify the report as a scoping review. | Title page | Reported in the manuscript title. |
| 2 | Structured summary | Provide a structured summary that includes, as applicable: background, objectives, eligibility criteria, sources of evidence, charting methods, results, and conclusions related to the review question and objectives. | Abstract | The abstract summarises the review aim, sources searched, synthesis approach, number of included studies, main findings, and conclusion. |
| 3 | Rationale | Describe the rationale for the review in the context of what is already known, and explain why a scoping review approach is appropriate. | Introduction | The Introduction describes the underuse of CR, uneven regional evidence, and the need to map availability, delivery, participation, and implementation barriers in WHO EMR countries. |
| 4 | Objectives | Provide an explicit statement of the questions and objectives being addressed with reference to their key elements. | End of Introduction | The objectives describe mapping CR availability, delivery models, participation metrics, barriers/enablers, and a Saudi Arabia-focused synthesis. |
| 5 | Protocol and registration | Indicate whether a review protocol exists; state if and where it can be accessed, and provide registration information where available. | Methods, Section 2.1 | The protocol is reported as registered with INPLASY, including the registration number and registration date. The manuscript states that registration occurred before formal database searches began. |
| 6 | Eligibility criteria | Specify characteristics of the sources of evidence used as eligibility criteria and provide a rationale. | Methods, Section 2.2 | Eligibility criteria are presented using the Participants-Concept-Context framework, including population, concept, context, evidence source types, timeframe, and language approach. |
| 7 | Information sources | Describe all information sources and the date the most recent search was executed. | Methods, Section 2.3; Supplementary Material 1 | Databases and regional sources are listed, with search dates and date coverage provided in the supplementary search strategy file. |
| 8 | Search | Present the full electronic search strategy for at least one database, including any limits used, so that it could be repeated. | Supplementary Material 1 | Full database-specific search strategies are provided for MEDLINE, Scopus, Web of Science Core Collection, CINAHL, Embase, and IMEMR. |
| 9 | Selection of sources of evidence | State the process for selecting sources of evidence, including screening and eligibility assessment. | Methods, Section 2.4 | The manuscript describes author-led screening with complete second-reviewer verification of title/abstract decisions and full-text eligibility decisions, with uncertainties resolved by discussion and rechecking against eligibility criteria. |

| Item | PRISMA-ScR section | Checklist item | Reported location | Comments / notes |
|---|---|---|---|---|
| 10 | Data charting process | Describe the methods of charting data from the included sources of evidence and any processes for verifying data. | Methods, Section 2.6 | A structured charting form was piloted and used; charted data for all included studies were independently verified by a second reviewer. |

## Supplementary Material 2. Completed PRISMA-ScR Checklist (continued)

| Item | PRISMA-ScR section | Checklist item | Reported location | Comments / notes |
|---|---|---|---|---|
| 11 | Data items | List and define all variables for which data were sought and any assumptions or simplifications made. | Methods, Section 2.6 | Extracted variables included bibliographic details, country, design, population, setting, objectives, delivery models, participation metrics, barriers/enablers, and related study characteristics. |
| 12 | Critical appraisal of individual sources of evidence | If done, provide a rationale for conducting a critical appraisal and describe the methods used. | Methods, Section 2.5 | Formal methodological quality assessment was not conducted, consistent with the scoping review objective. Key methodological characteristics were recorded to support interpretation. |
| 13 | Synthesis of results | Describe the methods of handling and summarising charted data. | Methods, Section 2.7 | The synthesis was descriptive and narrative, organised around country-level evidence, delivery models, participation metrics, barriers/enablers, and Saudi Arabia-specific findings. |
| 14 | Selection of sources of evidence | Give numbers of sources screened, assessed for eligibility, and included in the review, with reasons for exclusions at each stage, preferably using a flow diagram. | Results, Section 3.1; Figure 1 | The manuscript reports 108 records screened, 88 full texts assessed, 63 excluded, and 25 included; the PRISMA-ScR flow diagram is provided as Figure 1. |
| 15 | Characteristics of sources of evidence | For each source of evidence, present characteristics for which data were charted and provide citations. | Results, Section 3.2; Tables 1-2; Supplementary Table S1 | Study characteristics are summarised by country, design, sample, CR focus, and contribution in the main and supplementary evidence tables. |
| 16 | Critical appraisal within sources of evidence | If done, present data on critical appraisal of included sources. | Methods, Section 2.5; Discussion/Limitations | Not applicable as a formal quality appraisal was not performed; this is stated and justified in the Methods. |
| 17 | Results of individual sources of evidence | For each included source, present the relevant charted data related to the review questions and objectives. | Results, Sections 3.3-3.7; Tables 2-5; Supplementary Table S1 | Charted evidence is presented across programme characteristics, participation metrics, barriers/enablers, and Saudi Arabia-focused findings. |
| 18 | Synthesis of results | Summarise and/or present the charting results as they relate to the review questions and objectives. | Results, Sections 3.3-3.7; Tables 1-5 | Results are synthesised descriptively and narratively across availability, delivery models, participation metrics, barriers/enablers, and national Saudi findings. |
| 19 | Summary of evidence | Summarise the main results, including how they relate to the review questions and objectives | Discussion | The Discussion summarises that CR evidence in the WHO EMR is uneven and fragmented, with early losses at referral/enrolment and implementation barriers across multiple levels. |

| Item | PRISMA-ScR section | Checklist item | Reported location | Comments / notes |
| --- | --- | --- | --- | --- |
| 20 | Limitations | Discuss the limitations of the scoping review process. | Discussion, Limitations paragraph | Limitations include uneven evidence distribution, heterogeneous reporting of participation metrics, the primarily author-led process, lack of formal agreement statistics, and the evidence-mapping scope. |
| 21 | Conclusions | Provide a general interpretation of the results with respect to the review questions and objectives, and discuss implications for future research and practice. | Conclusion | The conclusion highlights fragmented evidence, limited translation into routine access, and priorities including referral strengthening, service capacity, and flexible delivery models. |
| 22 | Funding | Describe sources of funding for the included sources of evidence and for the scoping review; describe the role of funders. | Funding statement | The manuscript states that the review received no external funding. |
