# Supplementary material for: Cardiac Rehabilitation in the WHO Eastern Mediterranean Region: A Scoping Review with a Saudi Arabia–Focused Synthesis
Source: J Clin Med. 2026 Jun 7;15(12):4413. doi: 10.3390/jcm15124413 (PMC13300928; doi:10.3390/jcm15124413)
Supplement: Supplementary file 1 [file jcm-15-04413-s001.zip › Supplementary Material S2.pdf]

Supplementary Material 2. Full database-specific search strategies

Overview

The literature search was designed to identify studies on cardiac rehabilitation in countries of the World Health Organization Eastern Mediterranean Region (WHO EMRO), with emphasis on service availability, delivery models, participation pathways, and barriers and enablers to implementation and completion. Searches were conducted for studies published from 1 January 2000 to 14 January 2026. No language restrictions were applied at the search stage.

1. MEDLINE (via PubMed)

Date searched: 20 January 2026
Years covered: 1 January 2000 to 14 January 2026

Search string:

```
(
  "Cardiac Rehabilitation"[Mesh]
  OR "cardiac rehabilitation"[tiab]
  OR "exercise-based cardiac rehabilitation"[tiab]
  OR "cardiac rehab"[tiab]
  OR "cardiovascular rehabilitation"[tiab]
  OR "secondary prevention"[tiab]
)
AND
(
  "Coronary Artery Disease"[Mesh]
  OR "Myocardial Infarction"[Mesh]
  OR "Coronary Artery Bypass"[Mesh]
  OR "Acute Coronary Syndrome"[Mesh]
  OR "coronary artery disease"[tiab]
  OR CAD[tiab]
  OR "myocardial infarction"[tiab]
  OR MI[tiab]
  OR "post-MI"[tiab]
  OR "post myocardial infarction"[tiab]
  OR "coronary bypass"[tiab]
  OR "coronary artery bypass graft*"[tiab]
  OR CABG[tiab]
  OR "acute coronary syndrome"[tiab]
  OR ACS[tiab]
  OR "post-PCI"[tiab]
  OR "percutaneous coronary intervention"[tiab]
)
AND(service*[tiab]
```

```
  OR program*[tiab]
  OR programme*[tiab]
  OR delivery[tiab]
  OR model*[tiab]
  OR implementation[tiab]
  OR referral[tiab]
  OR uptake[tiab]
  OR enrolment[tiab]
  OR enrollment[tiab]
  OR adherence[tiab]
  OR completion[tiab]
  OR dropout[tiab]
  OR attrition[tiab]
  OR barrier*[tiab]
  OR enabler*[tiab]
  OR access[tiab]
  OR availability[tiab]
)AND
(
  "Saudi Arabia"[tiab] OR Riyadh[tiab] OR Jeddah[tiab] OR Mecca[tiab] OR
Makkah[tiab]
  OR "United Arab Emirates"[tiab] OR UAE[tiab] OR "Abu Dhabi"[tiab] OR
Dubai[tiab]
  OR Qatar[tiab] OR Doha[tiab]
  OR Bahrain[tiab] OR Manama[tiab]
  OR Kuwait[tiab] OR "Kuwait City"[tiab]
  OR Oman[tiab] OR Muscat[tiab]
  OR Yemen[tiab] OR Sanaa[tiab] OR "Sana'a"[tiab]
  OR Iraq[tiab] OR Baghdad[tiab]
  OR Jordan[tiab] OR Amman[tiab]
  OR Lebanon[tiab] OR Beirut[tiab]
  OR Syria[tiab] OR "Syrian Arab Republic"[tiab] OR Damascus[tiab]
  OR Palestine[tiab] OR "West Bank"[tiab] OR Gaza[tiab] OR Jerusalem[tiab] OR
Ramallah[tiab]
  OR Egypt[tiab] OR Cairo[tiab]
  OR Libya[tiab] OR Tripoli[tiab]
  OR Tunisia[tiab] OR Tunis[tiab]
  OR Algeria[tiab] OR Algiers[tiab]
  OR Morocco[tiab] OR Rabat[tiab]
  OR Sudan[tiab] OR Khartoum[tiab]
  OR Somalia[tiab] OR Mogadishu[tiab]
  OR Djibouti[tiab]
  OR Pakistan[tiab] OR Islamabad[tiab]
  OR Iran[tiab] OR Tehran[tiab]
  OR Afghanistan[tiab] OR Kabul[tiab])AND
("2000/01/01"[Date - Publication] : "2026/01/14"[Date - Publication])
```

Filters/limits applied:
No language restrictions were applied. Publication date was limited from 1 January 2000 to 14 January 2026. Geographic terms for WHO EMRO countries were incorporated into the search strategy using country names and selected capital-city terms where relevant.

Records retrieved:
n = 110

2. Scopus

Date searched: 22 January 2026
Years covered: 1 January 2000 to 14 January 2026

Search string:

```
TITLE-ABS-KEY(
  (
    "cardiac rehabilitation"
    OR "exercise-based cardiac rehabilitation"
    OR "cardiac rehab"
    OR "cardiovascular rehabilitation"
    OR "secondary prevention"
  )
  AND
  (
    "coronary artery disease"
    OR CAD
    OR "myocardial infarction"
    OR MI
    OR "post myocardial infarction"
    OR "acute coronary syndrome"
    OR ACS
    OR "coronary artery bypass graft*"
    OR CABG
    OR "percutaneous coronary intervention"
    OR PCI
    OR "post-PCI"
  )AND
  (
    service*
    OR program*
    OR programme*
    OR delivery
    OR model*
    OR implementation
    OR referral
    OR uptake
    OR enrolment
    OR enrollment
    OR adherence
    OR completion
    OR dropout
    OR attrition
    OR barrier*
    OR enabler*
    OR access
    OR availability
  )
  AND
  (
    "Saudi Arabia" OR Riyadh OR Jeddah OR Mecca OR Makkah
    OR "United Arab Emirates" OR UAE OR "Abu Dhabi" OR Dubai
    OR Qatar OR Doha
```

```
    OR Bahrain OR Manama
    OR Kuwait OR "Kuwait City"
    OR Oman OR Muscat
    OR Yemen OR Sanaa OR "Sana'a"
    OR Iraq OR Baghdad
    OR Jordan OR Amman
    OR Lebanon OR Beirut
    OR Syria OR "Syrian Arab Republic" OR Damascus
    OR Palestine OR "West Bank" OR Gaza OR Jerusalem OR Ramallah
    OR Egypt OR Cairo
    OR Libya OR Tripoli
    OR Tunisia OR Tunis
    OR Algeria OR Algiers
    OR Morocco OR Rabat
    OR Sudan OR Khartoum
    OR Somalia OR Mogadishu
    OR Djibouti
    OR Pakistan OR Islamabad
    OR Iran OR Tehran
    OR Afghanistan OR Kabul
  )
)
AND PUBYEAR > 1999
AND PUBYEAR < 2027
```

Filters/limits applied:
No language restrictions were applied. Publication years were limited from 2000 to 2026. Geographic terms for WHO EMRO countries were incorporated into the search strategy using country names and selected capital-city terms where relevant.

Records retrieved:
n = 108

3. Web of Science Core Collection

Date searched: 21 January 2026
Years covered: 1 January 2000 to 14 January 2026

Search string:

```
TS=(
  (
    "cardiac rehabilitation"
    OR "exercise-based cardiac rehabilitation"
    OR "cardiac rehab"
    OR "cardiovascular rehabilitation"
    OR "secondary prevention"
  )
  AND
  (
    "coronary artery disease"
    OR CAD
    OR "myocardial infarction"
    OR MI
    OR "post myocardial infarction"
    OR "acute coronary syndrome"
    OR ACS
    OR "coronary artery bypass graft*"
    OR CABG
    OR "percutaneous coronary intervention"
    OR PCI
    OR "post-PCI"
  )
  AND
  (
    service*
    OR program*
    OR programme*
    OR delivery
    OR model*
    OR implementation
    OR referral
    OR uptake
    OR enrolment
    OR enrollment
    OR adherence
    OR completion
    OR dropout
    OR attrition
    OR barrier*
    OR enabler*
    OR access
    OR availability
  )
  AND
```

```
  (
    "Saudi Arabia" OR Riyadh OR Jeddah OR Mecca OR Makkah
    OR "United Arab Emirates" OR UAE OR "Abu Dhabi" OR Dubai
    OR Qatar OR Doha
    OR Bahrain OR Manama
    OR Kuwait OR "Kuwait City"
    OR Oman OR Muscat
    OR Yemen OR Sanaa OR "Sana'a"
    OR Iraq OR Baghdad
    OR Jordan OR Amman
    OR Lebanon OR Beirut
    OR Syria OR "Syrian Arab Republic" OR Damascus
    OR Palestine OR "West Bank" OR Gaza OR Jerusalem OR Ramallah
    OR Egypt OR Cairo
    OR Libya OR Tripoli
    OR Tunisia OR Tunis
    OR Algeria OR Algiers
    OR Morocco OR Rabat
    OR Sudan OR Khartoum
    OR Somalia OR Mogadishu
    OR Djibouti
    OR Pakistan OR Islamabad
    OR Iran OR Tehran
    OR Afghanistan OR Kabul
  )
)
```

Filters/limits applied:
No language restrictions were applied. Publication years were limited from 2000 to 2026. Geographic terms for WHO EMRO countries were incorporated into the search strategy using country names and selected capital-city terms where relevant.

Records retrieved:
n = 108

---

4. CINAHL

Date searched: 21 January 2026
Years covered: 1 January 2000 to 14 January 2026

Search string:

```
TX(
  (
    "cardiac rehabilitation"
    OR "exercise-based cardiac rehabilitation"
    OR "cardiac rehab"
    OR "cardiovascular rehabilitation"
    OR "secondary prevention"
  )
```

```
AND
(
  "coronary artery disease"
  OR CAD
  OR "myocardial infarction"
  OR MI
  OR "post myocardial infarction"
  OR "acute coronary syndrome"
  OR ACS
  OR "coronary artery bypass graft*"
  OR CABG
  OR "percutaneous coronary intervention"
  OR PCI
  OR "post-PCI"
)
AND
(
  service*
  OR program*
  OR programme*
  OR delivery
  OR model*
  OR implementation
  OR referral
  OR uptake
  OR enrolment
  OR enrollment
  OR adherence
  OR completion
  OR dropout
  OR attrition
  OR barrier*
  OR enabler*
  OR access
  OR availability
)
AND
(
  "Saudi Arabia" OR Riyadh OR Jeddah OR Mecca OR Makkah
  OR "United Arab Emirates" OR UAE OR "Abu Dhabi" OR Dubai
  OR Qatar OR Doha
  OR Bahrain OR Manama
  OR Kuwait OR "Kuwait City"
  OR Oman OR Muscat
  OR Yemen OR Sanaa OR "Sana'a"
  OR Iraq OR Baghdad
  OR Jordan OR Amman
  OR Lebanon OR Beirut
  OR Syria OR "Syrian Arab Republic" OR Damascus
  OR Palestine OR "West Bank" OR Gaza OR Jerusalem OR Ramallah
  OR Egypt OR Cairo
  OR Libya OR Tripoli
  OR Tunisia OR Tunis
  OR Algeria OR Algiers
  OR Morocco OR Rabat
  OR Sudan OR Khartoum
  OR Somalia OR Mogadishu
```

```
    OR Djibouti
    OR Pakistan OR Islamabad
    OR Iran OR Tehran
    OR Afghanistan OR Kabul
  )
)
```

Filters/limits applied:
No language restrictions were applied. Publication years were limited from 2000 to 2026. Geographic terms for WHO EMRO countries were incorporated into the search strategy using country names and selected capital-city terms where relevant.

Records retrieved:
n = 108

5. Embase

Date searched: 22 January 2026
Years covered: 1 January 2000 to 14 January 2026

Search string:

```
(
  'cardiac rehabilitation'/exp
  OR 'cardiac rehabilitation':ti,ab,kw
  OR 'exercise-based cardiac rehabilitation':ti,ab,kw
  OR 'cardiac rehab':ti,ab,kw
  OR 'cardiovascular rehabilitation':ti,ab,kw
  OR 'secondary prevention':ti,ab,kw
)
AND
(
  'coronary artery disease'/exp
  OR 'myocardial infarction'/exp
  OR 'coronary artery bypass graft'/exp
  OR 'acute coronary syndrome'/exp
  OR 'coronary artery disease':ti,ab,kw
  OR cad:ti,ab,kw
  OR 'myocardial infarction':ti,ab,kw
  OR mi:ti,ab,kw
  OR 'post myocardial infarction':ti,ab,kw
  OR 'acute coronary syndrome':ti,ab,kw
  OR acs:ti,ab,kw
  OR 'coronary artery bypass graft*':ti,ab,kw
  OR cabg:ti,ab,kw
  OR 'percutaneous coronary intervention':ti,ab,kw
  OR pci:ti,ab,kw
  OR 'post-pci':ti,ab,kw
)
AND
(
  service*:ti,ab,kw
  OR program*:ti,ab,kw
  OR programme*:ti,ab,kw
  OR delivery:ti,ab,kw
  OR model*:ti,ab,kw
  OR implementation:ti,ab,kw
  OR referral:ti,ab,kw
  OR uptake:ti,ab,kw
  OR enrolment:ti,ab,kw
  OR enrollment:ti,ab,kw
  OR adherence:ti,ab,kw
  OR completion:ti,ab,kw
  OR dropout:ti,ab,kw
  OR attrition:ti,ab,kw
  OR barrier*:ti,ab,kw
  OR enabler*:ti,ab,kw
  OR access:ti,ab,kw
  OR availability:ti,ab,kw
)
```

```
AND
(
  'Saudi Arabia':ti,ab,kw OR Riyadh:ti,ab,kw OR Jeddah:ti,ab,kw OR
Mecca:ti,ab,kw OR Makkah:ti,ab,kw
  OR 'United Arab Emirates':ti,ab,kw OR UAE:ti,ab,kw OR 'Abu Dhabi':ti,ab,kw
OR Dubai:ti,ab,kw
  OR Qatar:ti,ab,kw OR Doha:ti,ab,kw
  OR Bahrain:ti,ab,kw OR Manama:ti,ab,kw
  OR Kuwait:ti,ab,kw OR 'Kuwait City':ti,ab,kw
  OR Oman:ti,ab,kw OR Muscat:ti,ab,kw
  OR Yemen:ti,ab,kw OR Sanaa:ti,ab,kw OR 'Sana''a':ti,ab,kw
  OR Iraq:ti,ab,kw OR Baghdad:ti,ab,kw
  OR Jordan:ti,ab,kw OR Amman:ti,ab,kw
  OR Lebanon:ti,ab,kw OR Beirut:ti,ab,kw
  OR Syria:ti,ab,kw OR 'Syrian Arab Republic':ti,ab,kw OR Damascus:ti,ab,kw
  OR Palestine:ti,ab,kw OR 'West Bank':ti,ab,kw OR Gaza:ti,ab,kw OR
Jerusalem:ti,ab,kw OR Ramallah:ti,ab,kw
  OR Egypt:ti,ab,kw OR Cairo:ti,ab,kw
  OR Libya:ti,ab,kw OR Tripoli:ti,ab,kw
  OR Tunisia:ti,ab,kw OR Tunis:ti,ab,kw
  OR Algeria:ti,ab,kw OR Algiers:ti,ab,kw
  OR Morocco:ti,ab,kw OR Rabat:ti,ab,kw
  OR Sudan:ti,ab,kw OR Khartoum:ti,ab,kw
  OR Somalia:ti,ab,kw OR Mogadishu:ti,ab,kw
  OR Djibouti:ti,ab,kw
  OR Pakistan:ti,ab,kw OR Islamabad:ti,ab,kw
  OR Iran:ti,ab,kw OR Tehran:ti,ab,kw
  OR Afghanistan:ti,ab,kw OR Kabul:ti,ab,kw
)
AND [2000-2026]/py
```

Filters/limits applied:
No language restrictions were applied. Publication years were limited from 2000 to 2026. Geographic terms for WHO EMRO countries were incorporated into the search strategy using country names and selected capital-city terms where relevant.

Records retrieved:
n = 100

6. WHO Index Medicus for the Eastern Mediterranean Region (IMEMR)

Date searched: 22 January 2026
Years covered: 1 January 2000 to 14 January 2026

Search string:

```
("cardiac rehabilitation" OR "cardiac rehab" OR "cardiovascular
rehabilitation" OR "secondary prevention")
AND
("coronary artery disease" OR "myocardial infarction" OR "acute coronary
syndrome" OR "coronary artery bypass graft" OR CABG OR PCI)
AND
(service* OR program* OR programme* OR delivery OR implementation OR referral
OR uptake OR enrolment OR enrollment OR adherence OR completion OR dropout OR
attrition OR barrier* OR enabler* OR access OR availability)
AND
("Saudi Arabia" OR Riyadh OR Jeddah OR Mecca OR Makkah
OR "United Arab Emirates" OR UAE OR "Abu Dhabi" OR Dubai
OR Qatar OR Doha
OR Bahrain OR Manama
OR Kuwait OR "Kuwait City"
OR Oman OR Muscat
OR Yemen OR Sanaa
OR Iraq OR Baghdad
OR Jordan OR Amman
OR Lebanon OR Beirut
OR Syria OR Damascus
OR Palestine OR Gaza OR Jerusalem OR Ramallah
OR Egypt OR Cairo
OR Libya OR Tripoli
OR Tunisia OR Tunis
OR Algeria OR Algiers
OR Morocco OR Rabat
OR Sudan OR Khartoum
OR Somalia OR Mogadishu
OR Djibouti
OR Pakistan OR Islamabad
OR Iran OR Tehran
OR Afghanistan OR Kabul)
```

Filters/limits applied:
No language restrictions were applied. Publication years were limited from 2000 to 2026. Geographic terms for WHO EMRO countries were incorporated into the search strategy using country names and selected capital-city terms where relevant.

Records retrieved:
n = 97

Hand-searching and forward citation tracking

Reference lists of all included studies were screened manually for additional eligible studies. Forward citation tracking was also undertaken for key included articles to identify any additional relevant records.

Additional records identified through hand-searching/citation tracking:

n = 0

Non-English records

No potentially eligible non-English full-text articles requiring translation were identified.

Number of non-English full-text records assessed:

n = 0

Number included after translation/assessment:

n = 0

Number excluded because translation was not feasible:

n = 0

Notes

Search strategies were adapted to the indexing structure and syntax requirements of each database. Controlled vocabulary terms and free-text keywords were combined as appropriate for each source.

Total records retrieved across databases before deduplication:
n = 631
