# Supplementary material for: Cardiac Rehabilitation in the WHO Eastern Mediterranean Region: A Scoping Review with a Saudi Arabia–Focused Synthesis
Source: J Clin Med. 2026 Jun 7;15(12):4413. doi: 10.3390/jcm15124413 (PMC13300928; doi:10.3390/jcm15124413)
Supplement: Supplementary file 1 [file jcm-15-04413-s001.zip › Supplementary Table S1.pdf]

Supplementary Table S1

No.	Study (short title)	Country/region	Year	Design	Sample	Main CR focus	Principal contribution
1	EMRO availability and delivery audit	EMRO region	2019	Cross-sectional regional audit	CR programmes across EMRO	Availability and delivery of CR services	Showed limited regional availability, low density, and capacity gaps
2	Saudi policymakers qualitative study	Saudi Arabia	2024	Qualitative interpretive descriptive	9 policymakers/leaders	Policy, implementation, uptake	Identified system-level Saudi barriers and enablers
3	Western Saudi outpatient CR barriers	Saudi Arabia	2023	Cross-sectional survey	141 respondents	Establishing outpatient/phase III CR	Highlighted limited availability, low awareness, workforce and guidance gaps
4	Saudi cardiologists KAP/referral survey	Saudi Arabia	2025	Cross-sectional online survey	140 cardiologists	Referral barriers after PCI	Showed moderate-good knowledge but low/variable referral
5	Saudi post-PCI patient barriers survey	Saudi Arabia	2024	Cross-sectional telephone survey	104 post-PCI patients	Enrolment barriers and secondary prevention adherence	Demonstrated very low referral and attendance; home-based CR preferred
6	Saudi post-CABG RCT	Saudi Arabia	2022	3-arm single-blind RCT	82 randomized	Home-based vs outpatient-based vs usual care	Home-based CR was effective and may sustain benefits
7	Saudi home-based CR RCT	Saudi Arabia	2012	RCT	49 post-CABG men	Home-based CR vs usual care	Demonstrated feasible and beneficial home-based CR
8	Abu Dhabi CR registry study	United Arab Emirates	2023	Retrospective registry-based study	1,774 attendees	PT-led outpatient CR	First detailed Gulf CR programme report with completion correlates

No.	Study (short title)	Country/region	Year	Design	Sample	Main CR focus	Principal contribution
						delivery and completion	
9	Qatar first CR programme	Qatar	2021	Retrospective cohort	682 enrolled	Engagement, completion, outcomes	High attendance/completion and meaningful risk-factor improvements
10	Qatar hybrid phase II CR	Qatar	2023	Quality improvement programme	96 enrolled, 43 completed hybrid	Hybrid CR during COVID-19	Feasible, safe, and cost-saving hybrid delivery
11	Lebanon DCE preferences study*	Lebanon	2023	Online discrete choice experiment	126 respondents	Preferences for rehabilitation package attributes	Supported eHealth/remote-support and patient-centred design
12	Lebanon cardiologists/cardiac surgeons KAP survey	Lebanon	2021	Cross-sectional KAP survey	83 respondents	Knowledge, attitudes, practices, referral barriers	Showed positive attitudes but substantial referral/system barriers
13	Iran provincial CR audit	Iran	2023	Cross-sectional audit sub-study	22 of 30 programmes	Availability, density, staffing, components	Showed concentration in capitals and large unmet need
14	Iran CR registry experience	Iran	2023	Registry methods/experience report	Isfahan outpatient CR registry	Registry architecture and phased CR workflow	Demonstrated centre-, home-, and hybrid-pathway monitoring
15	Yazd CR programme experience	Iran	2019	Descriptive programme report	Single programme report	Programme structure and participation metrics	Provided detailed real-world service data from a developing-country setting

No.	Study (short title)	Country/region	Year	Design	Sample	Main CR focus	Principal contribution
16	Iran physician referral factors	Iran	2011	Cross-sectional physician survey	122 cardiologists	Physician-related referral barriers	Showed limited access, education, and referral knowledge
17	West of Iran post-CABG utilisation	Iran	2014	Observational study	4,735 post-CABG patients	Referral, enrolment, completion, hybrid uptake	Showed low participation and benefit of systematic referral/hybrid models
18	Iran completion predictors	Iran	2015	Retrospective observational study	1,050 CR patients	Predictors of failure to complete	Highlighted sociodemographic and psychological determinants of dropout
19	Iran centre-based barriers / home-based attitudes	Iran	2021	Cross-sectional survey	204 CR-eligible patients	Barriers to centre-based participation	Strong support for structured home-based alternatives
20	Pakistan local attendance study	Pakistan	2012	Cross-sectional study	416 eligible; 151 attendees	Attendance at outpatient CR	Easier access associated with better uptake/completion
21	Pakistan qualitative home-based needs study	Pakistan	2025 VOR	Qualitative descriptive study	20 participants	Needs assessment for contextual home-based CR	Supported tailored, family-inclusive, low-literacy–sensitive home CR
22	Pakistan MCard trial	Pakistan	2022	RCT	160 post-ACS patients	mHealth-augmented CR	Improved quality-of-life outcomes and offered scalable adjunct care
23	Morocco physiotherapists survey	Morocco	2024	Cross-sectional electronic survey	145 physiotherapists	Knowledge, attitudes,	Highlighted training/resource barriers and strong

No.	Study (short title)	Country/region	Year	Design	Sample	Main CR focus	Principal contribution
						practices, barriers	professional willingness
24	Algeria first CR experience	Algeria	2008	Descriptive early programme report	First 158 coronary patients	Initial programme implementation and outcomes	Suggested feasibility of first-centre CR establishment
25	Iran sex-differences non-enrolment study	Iran	2023	Cross-sectional phone survey	1,053 referred non-attenders	Barriers to enrolment among non-enrollees	Showed greater barrier burden among women and need for tailored models
